# The Impact of *PLoS Pathogens*

**DOI:** 10.1371/journal.ppat.0030145

**Published:** 2007-09-28

**Authors:** John Young, Kasturi Haldar

**Image 1:**
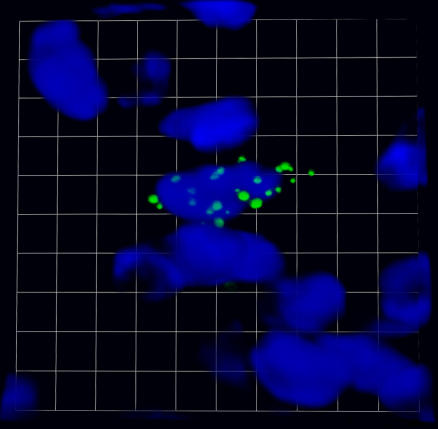
XMRV-infected stromal cells are detected in prostate tumor sections by fluorescence in situ hybridization. Green represents XMRV nucleic acid, and blue represents DAPI-stained nuclei. Credit: Photo by Ross J. Molinaro.

As we approach the second anniversary of the launch of *PLoS Pathogens*, it is evident that the journal has rapidly established itself as an important publication in the field of pathogen–host interactions (Image 1). We attribute this success to the commitment and motivation of our talented editorial board, to a receptive community (Table 1) who sees this as a leading open-access journal, and to a highly competent and helpful staff at the Public Library of Science.

**Table 1:**
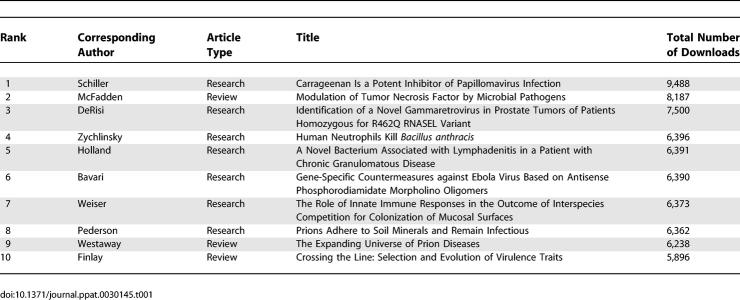
Top Ten Downloaded Papers from *PLoS Pathogens*, September 2005 to July 2007

Our initial impact factor, recently assigned by Thomson Scientific (formerly Thomson ISI), is 6.056. For an initial metric calculated from just four months of data, this is a strong start, and we fully expect it to rise rapidly as more information becomes available.

But, we also caution about reading too much into impact factor values because of inherent flaws that exist with using this number to measure the impact of any given paper. We recognize that journal impact factors are so often used as a surrogate measure of the quality of a given scientist and his or her work, influencing hiring, promotion, and even grant funding decisions, and for these reasons we think it is important to raise awareness about what impact factors measure.

The journal impact factor for any given year is calculated based on information obtained from the preceding two years. For example, for 2007:

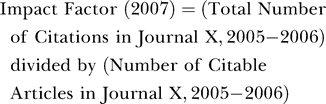
It is self-evident that this formula fails to reveal the significance of any given paper. Instead, the calculated number represents an average number of citations for a paper in the journal. In other words, the highest impact papers are given an artificially low ranking by this system, whereas papers with the least impact are assigned a greater impact value than they deserve. Confounding this problem, review articles, which are highly visible and citable, artificially inflate the “impact” of the lesser-cited research articles in the same journal. Also, the method by which “citable articles” are calculated in the denominator of this equation is unclear and needs to be made transparent so that the community can become more confident about the nature of the differences that exist between the impact of distinct journals. Clearly, a more scientifically rigorous methodology must be developed if we are to accurately quantify the true impact of a given paper. Several alternative mechanisms have been proposed, including measuring the number of downloads of a paper over the Internet, and we will not elaborate further on these ideas here. However, if you would like to learn more, we encourage you to read the articles cited at the end of this editorial [1–4].

In closing, we are grateful to you for establishing *PLoS Pathogens* as a leading open-access journal. On behalf of the editorial board we thank you, the community, for your confidence in the journal and for your continued support.
